# Self-supervised learning reduces label noise in sharp wave ripple classification

**DOI:** 10.1038/s41598-025-90380-x

**Published:** 2025-03-05

**Authors:** Saber Graf, Pierre Meyrand, Cyril Herry, Tiaza Bem, Feng-Sheng Tsai

**Affiliations:** 1 Neurocentre Magendie, INSERM U1215, University Bordeaux, Bordeaux, France; 2 Nalecz Institute of Biocybernetics and Biomedical Engineering, Polish Academy of Sciences, Warsaw, Poland; 3 Department of Biomedical Informatics, China Medical University, Taichung, 40402 Taiwan; 4 Research Center for Interneural Computing, China Medical University Hospital, Taichung, 40447 Taiwan

**Keywords:** Label noise, Self-supervised learning (SSL), Sharp wave ripples (SWRs), Time-series data classification, Neuroscience, Mathematics and computing

## Abstract

In the field of electrophysiological signal analysis, the classification of time-series datasets is essential. However, these datasets are often compromised by the prevalent issue of incorrect attribution of labels, known as label noise, which may arise due to insufficient information, inappropriate assumptions, specialists’ mistakes, and subjectivity, among others. This critically impairs the accuracy and reliability of data classification, presenting significant barriers to extracting meaningful insights. Addressing this challenge, our study innovatively applies self-supervised learning (SSL) for the classification of sharp wave ripples (SWRs), high-frequency oscillations involved in memory processing that were generated before or after the encoding of spatial information. This novel SSL methodology diverges from traditional label correction techniques. By utilizing SSL, we effectively relabel SWR data, leveraging the inherent structural patterns within time-series data to improve label quality without relying on external labeling. The application of SSL to SWR datasets has yielded a 10% increase in classification accuracy. While this improved classification accuracy does not directly enhance our understanding of SWRs, it opens up new pathways for research. The study’s findings suggest the transformative capability of SSL in improving data quality across various domains reliant on precise time-series data classification.

## Introduction

The analysis of time-series data, especially derived from electrophysiological studies, has emerged as a pivotal element in the comprehensive understanding of complex neural processes^1^. A central challenge in this domain is the accurate classification of data, particularly when dealing with noisy or inconsistent labels. Recent studies have highlighted how label noise can significantly impair model performance in physiological signal classification tasks, with impacts ranging from decreased accuracy to altered class distributions that affect clinical interpretations^2^.

Label noise in time-series data is particularly challenging due to the temporal dependencies and sequential patterns inherent in these signals^3^. In physiological recordings, this noise often stems from experimental variability, subjective interpretation of signals, and the inherent complexity of biological systems. The impact is multifaceted, affecting not only classification accuracy but also feature selection and model training processes^4^. Traditional approaches to handling label noise, such as data cleansing or noise-robust models, often fall short when applied to time-series data, as they can disrupt temporal relationships or lead to the loss of valuable information^5^.

The growing complexity of physiological recordings, coupled with the increased adoption of automated analysis methods, has made the challenge of label noise even more pressing. High-density neural recordings and continuous monitoring systems generate vast amounts of data that require reliable classification, yet the process of obtaining accurate labels becomes increasingly challenging at scale^6^. This has led to a critical need for methods that can handle label noise while preserving the complex temporal patterns essential for understanding biological processes.

Sharp wave ripples (SWRs) in hippocampal recordings present a particularly challenging case for time-series classification. These brief, high-frequency oscillatory patterns play a crucial role in memory consolidation^7^, making their accurate classification essential for understanding neural processes. SWRs reflect firing and synaptic current sequences emerging from cognitively relevant neuronal ensembles, but their classification is complicated by biological variability, recording conditions, and the subtle nature of learning-related changes^8^.

Despite recent progress in deep learning approaches for SWR detection^6^, the challenge of label noise remains a significant barrier to reliable classification. Traditional supervised learning methods struggle with the inherent variability in SWR patterns and the difficulty of obtaining consistent labels across different experimental conditions^9^. This has motivated the exploration of new approaches that can better handle the uncertainty and noise inherent in physiological recordings.

In this work, we introduce a novel self-supervised learning (SSL) based approach that specifically addresses the challenges of label noise in physiological time-series classification. Building on recent advances in temporal representation learning^10^, we develop a framework that combines temporal contrasting with robust label correction mechanisms. Our approach innovatively adapts recent developments in contrastive learning and temporal modeling to handle both temporal dependencies and label noise while preserving the essential characteristics of the physiological signals.

The effectiveness of our approach is validated across multiple physiological datasets beyond SWRs, including ECG arrhythmia detection and epileptic seizure recognition. Through extensive experiments, we demonstrate significant improvements in classification accuracy. Our method shows remarkable generalization capabilities across different types of physiological signals while requiring minimal manual intervention, addressing key limitations of existing approaches.

## Related works

The challenge of label noise in machine learning has been extensively studied, with early work by Garcia et al.^4^ establishing its fundamental effects on model complexity and performance. Traditional approaches to handling label noise have evolved along several paths. Label Noise-Robust Models^11^ aim to build inherent resilience into learning algorithms through techniques like model bagging and ensemble methods. Data cleansing approaches focus on identifying and removing mislabeled instances through statistical methods and anomaly detection^5^, while noise-tolerant learning algorithms adapt their mechanisms to accommodate noise through probabilistic modeling. However, as shown by Ding et al.^2^, these approaches often struggle with the complex temporal dependencies inherent in physiological signals.

The emergence of self-supervised learning (SSL) has opened new possibilities for handling label noise in time-series data. Recent comprehensive surveys by Zhang et al.^12^ highlight how SSL can leverage unlabeled data to learn robust representations while reducing dependence on potentially noisy labels. The field has seen rapid development in both contrastive and generative approaches, with Liu et al.^13^ demonstrating their comparative effectiveness in time-series applications. Notably, Vaaras et al.^14^ introduced PFML, showing improved robustness against representation collapse in temporal data, while Eldele et al.^10^ proposed a temporal contrasting framework specifically addressing the challenges of learning from time-series with limited labeled data.

In physiological signal processing, SSL has shown particular promise. Zou et al.^15^ demonstrated the effectiveness of combining LSTM networks with contrastive learning for EEG classification, while Senane et al.^16^ introduced diffusion-based approaches showing superior performance in handling complex physiological patterns. These advances have proven especially relevant for clinical applications, where data quality issues often compromise traditional supervised approaches. The development of foundation models for time-series analysis^17^ has further enhanced SSL’s potential in physiological signal processing, demonstrating strong transfer learning capabilities crucial for scenarios with noisy or limited labeled data.

In the specific domain of Sharp Wave Ripple classification, recent work has highlighted both the challenges and opportunities in applying modern machine learning approaches. Navas-Olive et al.^18^ demonstrated how deep learning models can effectively capture both local and global temporal patterns in SWR signals, while Hsu et al.^9^ showed the potential for distinguishing learning-related alterations in hippocampal ripples. However, these approaches still face challenges in handling label noise, particularly given the subtle temporal patterns characteristic of SWRs.

The integration of temporal-aware SSL approaches with physiological signal classification represents a promising direction for improving robustness to label noise while maintaining sensitivity to complex temporal patterns. Our work builds upon these advances while addressing several key limitations in existing approaches, particularly in preserving critical temporal relationships in physiological time-series data.

## Results

We are building upon the research described in Hsu et al. 2021^9^, where convolutional neural networks (CNNs) were used to categorize sharp wave-ripple (SWR) events generated in mice’s hippocampus as occurring either before or after a learning task in a maze. Hsu et al. 2021 reported a classification accuracy of 66.29%. This level of accuracy, while significant, prompted the research undertaken in our paper, aiming to improve SWR classification through new methodologies.

### Data acquisition and preparation

**Fig. 1:**
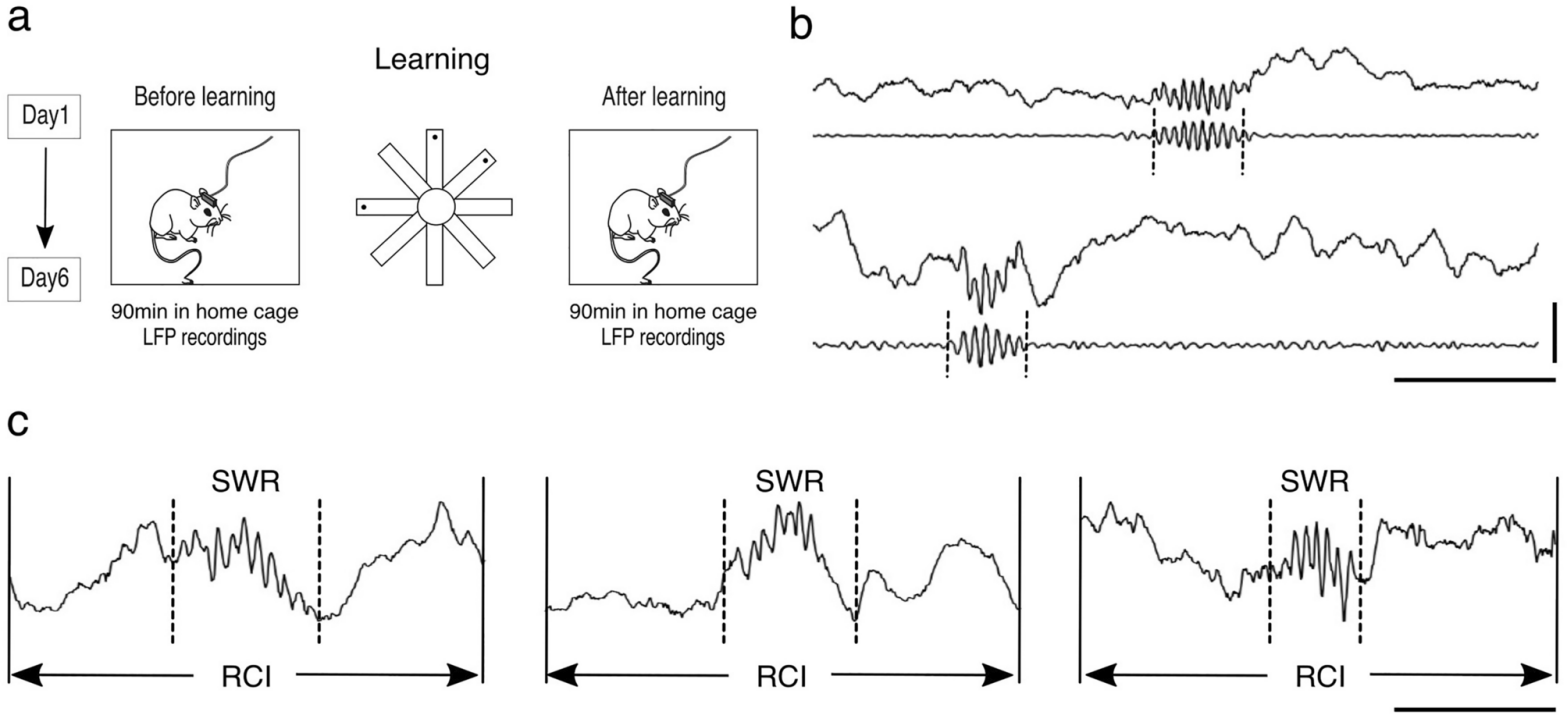
SWR dataset preparation: recording, detection, and segmentation process. (**a**) Schematic of the experimental design, indicating neural data collection from mice before and after learning tasks in a radial maze. (**b**) Raw (upper traces) and filtered (bottom traces) electrophysiological signals showing detected SWRs from CA1 hippocampal recording. (**c**) Segmentation of local field potentials into non-overlapping ripple-centered intervals (RCIs), prepared for deep learning classification process. Dashed vertical lines indicated the onset and offset of the SWR. Horizontal scale 100 ms, vertical scale 1 mV.

In this study, we employed the SWR dataset, the same dataset that was utilized in a previous work^9,19^. The behavioral experiment involved mice navigating in the eight-arm radial maze, a task designed to study spatial learning and memory formation, while electrophysiological signals were recorded from the dorsal hippocampus CA1 region before and after the trials (Fig. 1a).

Subsequent to the behavioral experiments, SWR detection was performed from the recorded local field potential (LFP) signals (Fig. 1b, top traces). These signals were initially captured at a 40 kHz sampling rate and subsequently down sampled to 2,000 Hz for analysis. The process involved the application of a 4th-order Chebyshev Type II filter, accompanied by the Hilbert transformation to compute the signal envelope, which determined the onset and offset of SWR events (Fig. 1b, bottom traces (see dashed vertical lines)).

In preparation for deep learning classification, we segmented the LFP signals into non-overlapping ripple-centered intervals (RCIs) (Fig. 1c). Each RCI represents a data segment of 256 ms, corresponding to 512 data points. This interval length was chosen to ensure inclusion of SWR events of variable durations and their associated pre- and post-ripple neural activities, which can contain important information. Moreover, maintaining a consistent input size is essential for the training of deep learning models.

It is pivotal to clarify that, throughout this text, the terms SWRs and their corresponding segmented intervals, RCIs, are used interchangeably. This is because our analytical focus is on RCIs, segments centered around SWRs.

### Benchmarking deep learning algorithms

We first tested a variety of deep learning models in order to check if their performances were better than that of the CNN model described in Hsu et al. 2021. Our approach involved two distinct processing methods: employing 1D models to handle raw SWR temporal sequences directly and using 2D models where the SWRs were first converted into images and then used as inputs to the models. The 2D models tested included MobileNet^20^, EfficientNetB0^21^, VGG16^22^, and ConvNeXt^23^ architectures renowned for their image classification capabilities. For the 1D temporal data, we utilized LSTM^24^ and 1D CNN^25^ models, which are traditionally tailored for sequential data.

**Fig. 2:**
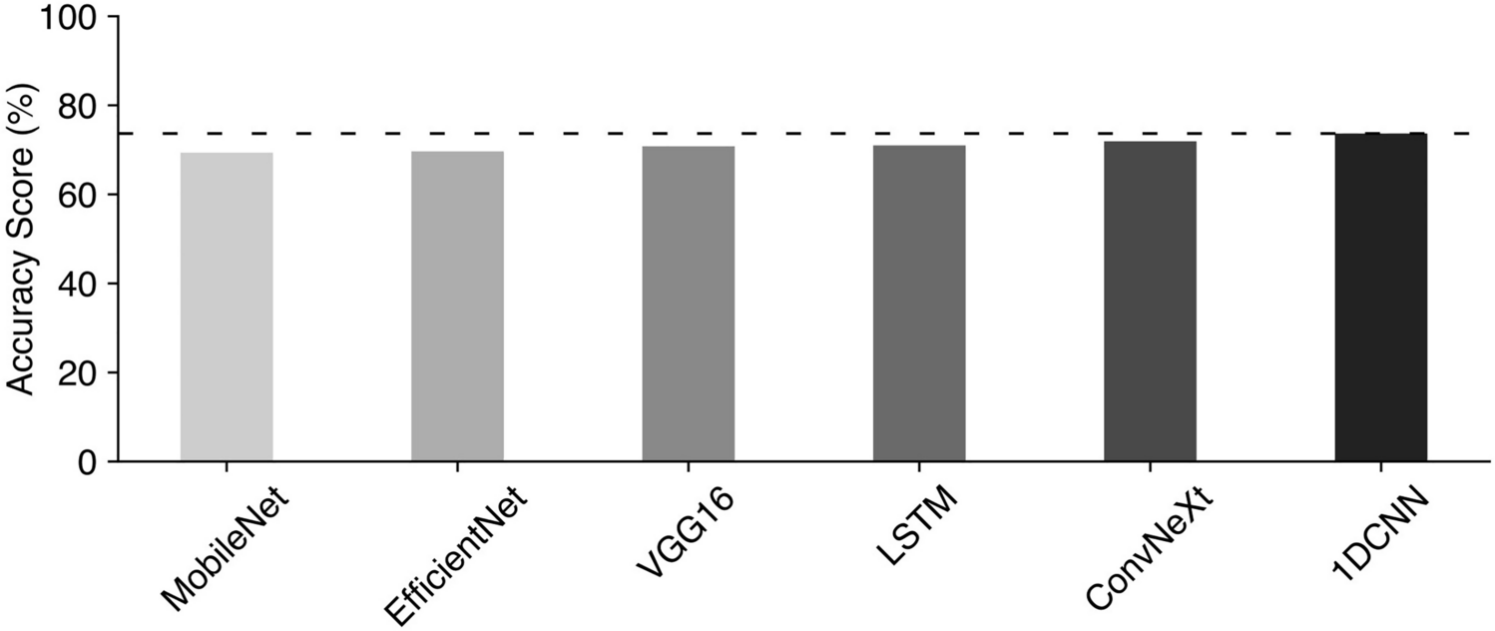
Benchmarking deep learning algorithms used for classification of SWRs. The lowest accuracy score was achieved using MobileNet, whereas the highest accuracy was observed with 1D CNN. Notice however relatively flat distribution of performance level among all tested models.

Through our benchmarking efforts, it became evident that the accuracy scores of the various models were tightly grouped, achieving between 69% and 73% accuracy (Fig. 2), with the 1D CNN model, particularly after hyperparameter optimization, achieving the upper limit of this accuracy range (Fig. 2, darkest bar). On the other hand, the modest improvements across all models prompt us to consider underlying issues in our data, specifically the possibility of label noise, referring to inaccuracies or inconsistencies in the assigned labels of SWRs, where events categorized as before learning or after learning may not accurately reflect the true state due to biological variability or errors in labeling.

### Addressing label noise

The labeling of SWRs as ‘before’ or ‘after’ based on their occurrence relative to the learning task in a maze experiment was initially straightforward. However, as the animals underwent repeated experimental conditions over six days, a suspicion arose that a given SWR generated before learning task could express features gained during the previous experimental day(s). Indeed, a potential activation of previously formed memories before learning session complicates the classification, as it could produce SWRs that closely resemble those observed after the learning phase. Additionally, the inherent nature of some of the SWRs, which may not be significantly altered by the learning task, could contribute to this challenge since some SWRs generated after session may resemble those generated before the session. These possibilities likely hamper the models’ ability to learn and differentiate effectively between the two classes. We therefore initiated efforts to reconsider our dataset’s labels. We claimed that the application of self-supervised learning (SSL) with contrastive learning was effective in enhancing classification performance. By leveraging the contrastive loss to bring similar instances closer and push dissimilar instances apart in the feature space, thus realigning our labels to reflect the data’s intrinsic structure more accurately. This realignment effectively refines the labeling, which in turn reduces label noise. Finally, dataset characterized by improved integrity due to the reduction of label noise which are used during training the 1D CNN model should lead to enhanced classification outcomes.

### Implementation of SSL re-labeling

**Fig. 3:**
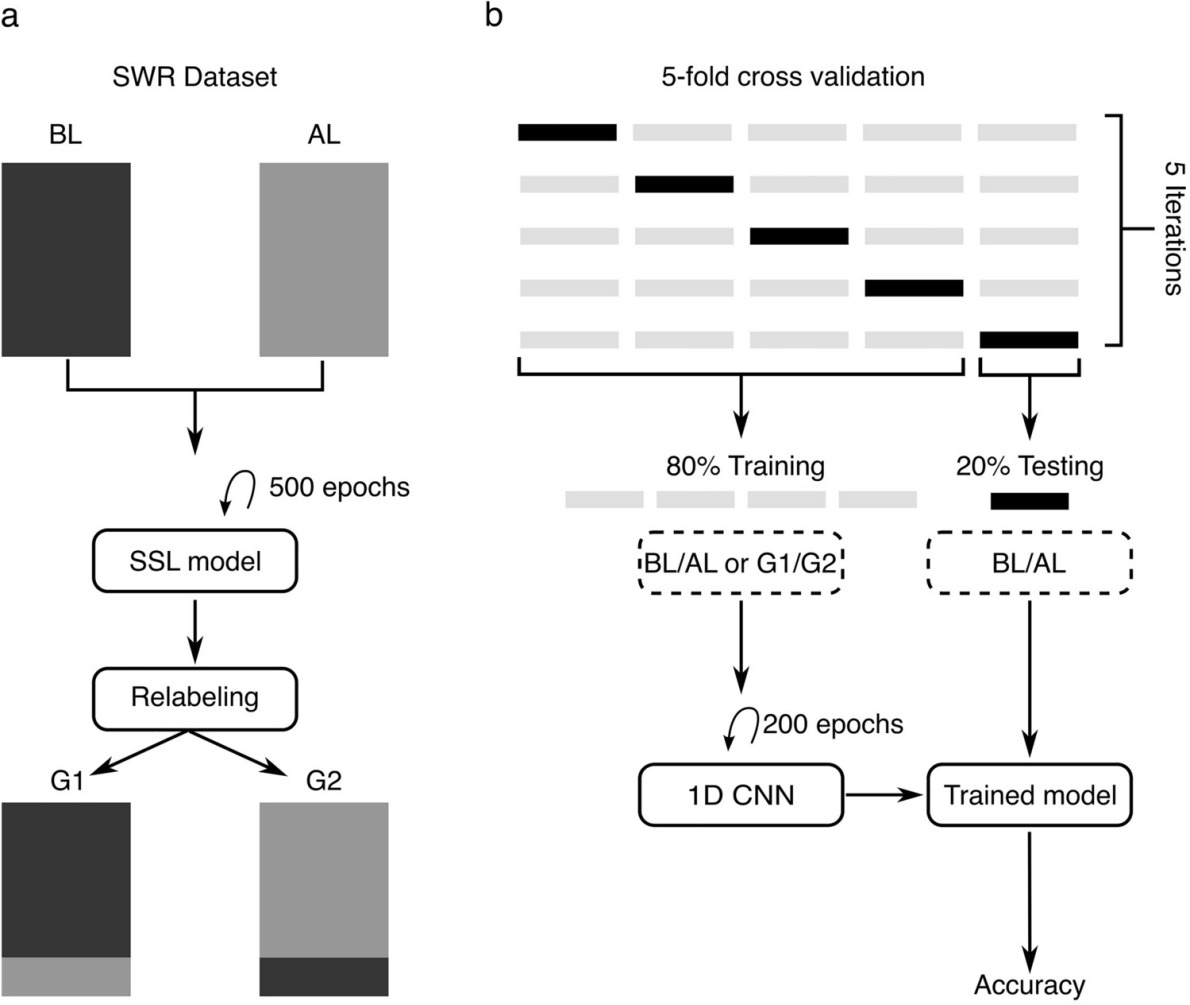
SSL re-labeling and 1D CNN training paths. (**a**) The SSL model re-labels the SWR dataset, categorizing before-learning (BL) and after-learning (AL) instances into new groupings, Group 1 (G1) and Group 2 (G2), based on similarities in features. (**b**) In each iteration of a 5-fold cross-validation, 80% of the data is allocated to two distinct training sets: with original labels (BL/AL) or re-labeled (G1/G2), and 20% with original BL/AL labels is used for testing. The 1D CNN is then trained over 200 epochs, which is followed by an evaluation phase where the model’s accuracy is assessed using the test data.

We implemented our SSL re-labeling method, drawing inspiration from the Time-Series Representation Learning via Temporal and Contextual Contrasting (TS-TCC) framework^26^. During the 500-epoch training, the SSL model was trained on the SWR dataset, comprising instances before learning (BL) and after learning (AL), without any labels (Fig. 3a). Following this training period, the model utilized its newly refined features, acquired through data augmentation and contrastive learning (see Materials and methods), to generate new labels for our dataset. These labels were designated as Group 1 and 2 (G1 and G2), based on the proximity of features evaluated by the model (Fig. 3a).

Subsequent to SSL re-labeling, we initiated the training of a 1D CNN model, employing a 5-fold cross-validation method to evaluate the model’s accuracy in two alternative ways, utilizing either the original BL/AL labels or the G1/G2 labels newly generated by the SSL (Fig. 3b). For each cross-validation iteration, 80% of the dataset is designated for training whereas the remaining 20%, always labeled with the original BL/AL, was consistently used for testing. This ensured that the model’s performance was assessed against a standard label set. The training was conducted for 200 epochs in each iteration (an epoch refers to one complete cycle through the full training dataset), with each iteration aiming to enhance the robustness and generalizability of the model’s performance (Fig. 3b).

**Fig. 4:**
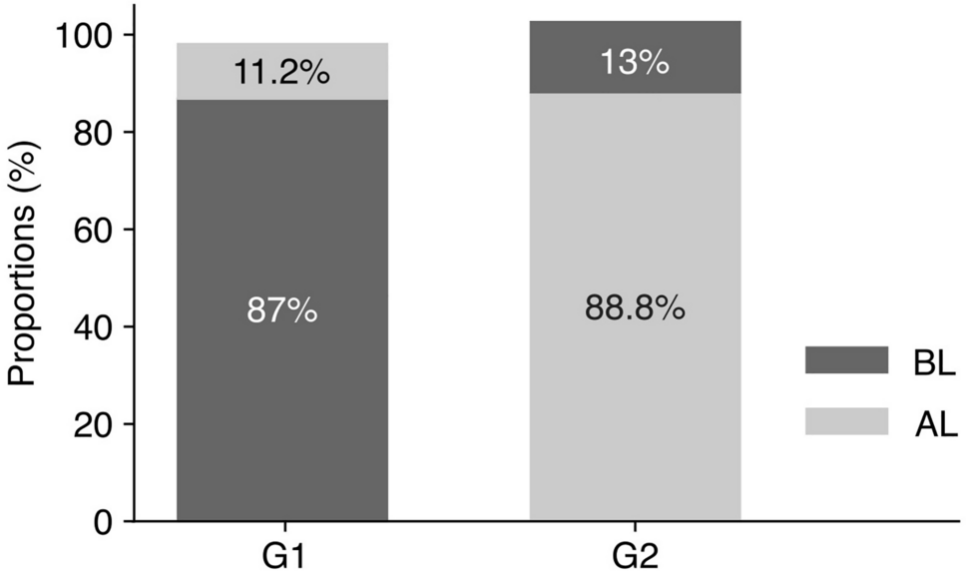
Distribution of SWRs after SSL re-labeling. The bar graph shows the proportion of SWRs recorded before learning (BL) and after learning (AL) within the newly established groups G1 and G2.

Applying the SSL model to our SWR dataset resulted in a new distribution of the SWRs into two distinct groups. Whereas Group 1 (G1) comprised 87% of the SWRs identified as before learning (BL) and 11.2% identified as after learning (AL) (Fig. 4, left bar), Group 2 (G2) contained 88.8% AL SWRs and 13% BL SWRs (Fig. 4, right bar). This balance in re-labeling indicates a methodical approach that does not skew the dataset in favor of either class.

We postulated that relabeling of the dataset will lead to a more robust and precise classification outcome.

### 1D CNN model performance

**Fig. 5:**
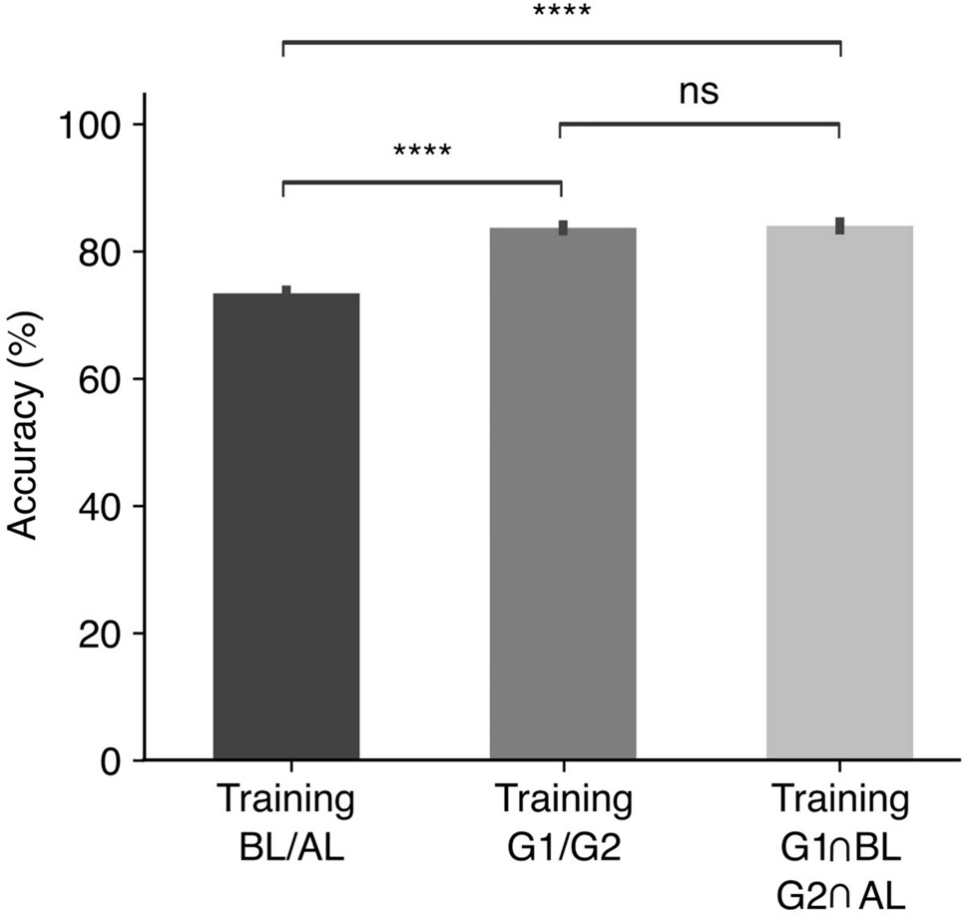
Impact of SSL re-labeling on 1D CNN model accuracy. Accuracy of the 1D CNN model trained with the re-labeled data (G1/G2) (middle bar) or trained on the intersection of G1 with BL and G2 with AL (right bar) is significantly increased compared to the 1D CNN model trained with original data set (BL/AL) (left bar). All tests were done on BL/AL. Statistical significance, determined via t-tests, is marked directly above the bars: ****$p> 0.0001$, and ‘ns’ for non-significance with $p> 0.05$. Vertical lines indicate the standard error of the mean (SEM).

To evaluate the impact of SSL re-labeling on classification accuracy, an initial training of the 1D CNN model was conducted using the original dataset labels (before learning, BL; after learning, AL). This preliminary phase resulted in a model accuracy of 73.28% (Fig. 5, left bar). Upon re-labeling the dataset through the SSL approach, the 1D CNN model exhibited a significant increase in classification accuracy, registering at 83.66% (N = 5, t-test, $p>0.0001$) (Fig. 5, middle bar).

Further analysis was conducted by examining the intersection of SSL re-labeled groups with the original labels-specifically, the intersection of Group 1 (G1) with BL instances and Group 2 (G2) with AL instances. When the 1D CNN model was trained on this intersected dataset, the accuracy achieved was also significantly higher than that using original dataset labels and expressed 84.11% (N = 5, t-test, $p>0.0001$) (Fig. 5, right bar). Moreover, the training on the intersected groups ($G1 \cap BL, G2 \cap AL$) resulted in a similar level of performance as the training on G1 and G2 (N = 5, t-test, $p>0.05$).

### Comparison with similar time-series label noise methods

To evaluate our approach in the context of existing time-series label noise solutions, we implemented and compared our SSL method with two recent approaches: Self-Re-Labeling with Embedding Analysis (SREA)^27^ and Confident Time-Warping (CTW)^28^. Both methods, like ours, specifically address the challenges of label noise in time-series data. All methods were evaluated on the same SWR dataset using identical train/test splits and evaluation protocols (see Table 1).

**Table 1:** Performance comparison of different label noise handling approaches on the SWR dataset.

Method	Accuracy (%)	F1-Score	Precision	Recall
SREA	76.23 ± 2.11	0.75	0.77	0.76
CTW	74.52 ± 6.83	0.73	0.75	0.74
Our SSL	**84.11** ± **1.20**	**0.83**	**0.84**	**0.84**

SREA achieved a mean accuracy of 76.23% (±2.1%) across five runs. While this represents an improvement over the baseline CNN model (73.28%), we observed performance degradation in later epochs, suggesting potential over-correction of labels. CTW showed more variable performance, with accuracy scores ranging from 69.81% to 83.33% (mean 74.52% ± 6.8%), indicating challenges in maintaining stable performance across different runs.

Our SSL approach achieved 84.11% (±1.2%) accuracy with notably lower variance across runs. The smaller standard deviation in our results (±1.2% compared to ±6.8% for CTW) suggests better stability and robustness to different initializations and data splits. The balanced precision and recall scores further indicate consistent performance across all evaluation metrics.

### Testing the role of label noise

**Fig. 6:**
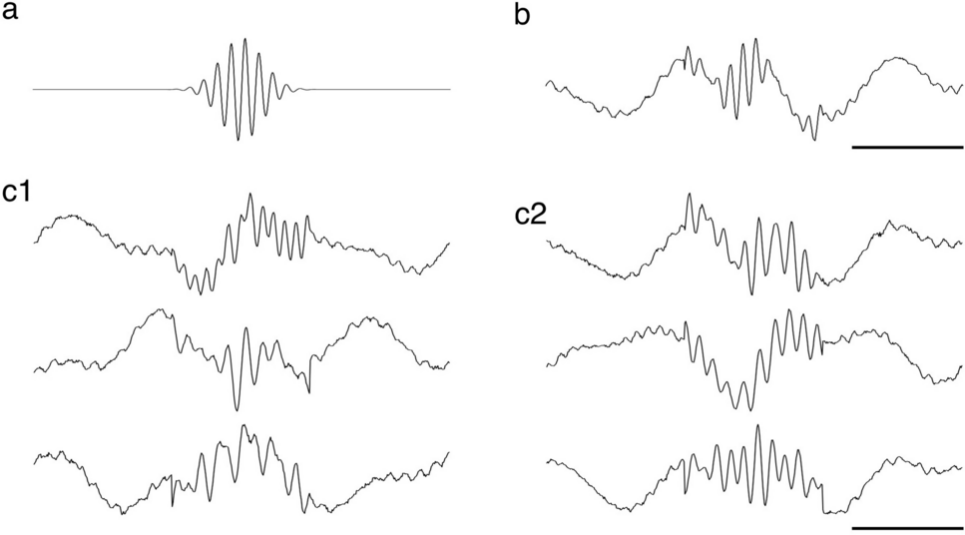
Construction of the synthetic ${SWR}_{art}$ dataset. (**a**) Gaussian-modulated sinusoidal wave simulates the core oscillatory pattern of SWR events at a frequency of 120–200 Hz. (**b**) A single RCI is derived from the initial model by adding delta (1–3 Hz) and low gamma (20–40 Hz) oscillations, complemented by Gaussian white noise with a mean of zero and a variable standard deviation. Examples of ${\text{SWR}}_{art}$ representing SWR not affected by learning (**c1**) and transformed by encoded information (**c2**). Horizontal scale 100 ms.

To test the role of different levels of label noise, we conducted a series of experiments on an artificial sharp wave-ripple (${\text{SWR}}_{art}$) dataset. Each ${\text{SWR}}_{art}$ was initially modeled using a Gaussian-modulated sinusoidal wave with a frequency between 120 and 200 Hz, reflecting the intrinsic frequency observed in naturally occurring SWRs^19^. This sine wave underwent Gaussian modulation, echoing the distinct rise and fall of the amplitude of SWRs captured in vivo (Fig. 6a). The ${\text{SWR}}_{art}$ instances were designed to have a random duration, normally distributed between 150 to 200 points, to introduce variability. For each ${\text{SWR}}_{art}$, we added signal segments before and after the SWR to form a RCI of 512 points. This ensured that our samples always had the same size, which is required for our deep learning models.

To introduce more biological realism, we incorporated delta (1–3 Hz) and low gamma oscillations (20–40 Hz)^8^ present in the hippocampal region during slow wave sleep^7^, using a normal distribution to randomly select a frequency within these ranges, and white noise to ${\text{SWR}}_{art}$ as well as to entire RCIs (Fig. 6b). We generated 20,000 ${\text{SWR}}_{art}$ belonging to two different classes, namely Class 1 which contained ${\text{SWR}}_{art}$ representing SWRs not affected by learning and Class 2 comprising ${\text{SWR}}_{art}$ which represented SWRs transformed by learning. The Class 1 and 2 samples were constructed using different ranges of frequency, amplitudes, and noise levels (see Table 2). In Class 1 the ${\text{SWR}}_{art}$ frequency was modulated to span a range from 0.8 to 1.2 times base frequency, following a normal distribution. The base frequency was set at 160 Hz that is the midpoint within the observed frequency range of natural SWRs (120–200 Hz). Also, the amplitude in Class 1 fluctuated between 0.8 to 1.2 times a standardized base amplitude, following a normal distribution. This base amplitude was defined as a range between −1 and 1 in the simulation model to ensure uniformity across different simulations. The noise level in these samples incorporated Gaussian white noise with a mean of zero and a standard deviation of 0.1, empirically derived from the standard deviation of noise measured in biological SWR recordings.

**Table 2:** Multiplication factors of the frequency, amplitude, and noise level used for construction of ${\text{SWR}}_{art}$. Base frequency: 160 Hz; Base amplitude: a range between −1 and 1; Base noise level (standard deviation): 0.1.

Multiplication factors	Frequency	Amplitude	Noise
Class 1	0.8–1.2	0.8–1.2	1–1.5
Class 2	1.1–1.3	0.95–1.05	0.5–1

Conversely, the Class 2 was constructed with a marked reduction in both variability of frequency and amplitude, as well as noise levels presumably imitating the neural activities characterizing the post learning state. This was quantitatively reflected in the frequency ranging of 1.1 to 1.3 times the base frequency, amplitude ranging from 0.95 to 1.05 times the base amplitude, and the noise level with a standard deviation ranging from 0.5 to 1 time the base noise level (standard deviation) (Table 2).

### Assessing of SSL re-labeling efficacy using the synthetic ${\text{SWR}}_{art}$ dataset

To examine the robustness of SSL re-labeling approach, we incorporated the ${\text{SWR}}_{art}$ dataset into our validation process. This stage specifically aimed to assess the SSL model’s capability to accurately adjust labels within a controlled setup, where various proportions of Class1 and Class 2 ${\text{SWR}}_{art}$ were used.

First, in order to mimic biological diversity, artificial before learning $(\hbox {BL}_{art})$ and after learning $(\hbox {AL}_{art})$ groups were constructed using ${\text{SWR}}_{art}$ belonging to Class 1 and Class 2 in the proportion varying from 0 to 50% thereafter referred as “label noise”. For example, for the proportion 20% whereas the group $\hbox {BL}_{art}$ comprised 80% of Class 1 and 20% of Class 2, the group $\hbox {AL}_{art}$ contained 20% of Class 1 and 80% of Class 2. Second, the SSL model was employed to generate new labels, thus creating the $\hbox {G1}_{art}$ and $\hbox {G2}_{art}$ groups.

Third, similarly to the previously described biological SWR classification, this adjusted dataset was processed in the training in two distinct streams: one without the application of SSL ($\hbox {BL}_{art}$ and $\hbox {AL}_{art}$) and the other with SSL re-labeling ($\hbox {G1}_{art}$ and $\hbox {G2}_{art}$). Testing was always processed on $\hbox {BL}_{art}$ and $\hbox {AL}_{art}$. A 5-fold cross-validation was used, systematically dividing the dataset into an 80% subset for training and a 20% subset for testing. This division was crucial to ascertain the robustness of the 1D CNN model’s learning across folds, ensuring that the model was exposed to diverse segments of the data for both training and validation. The performance of the 1D CNN model was evaluated on the test subset of $\hbox {BL}_{art}$ and $\hbox {AL}_{art}$ data. This final evaluation served as a quantitative assessment of the model’s capability to correctly classify ${\text{SWR}}_{art}$.

### Evaluating the impact of label noise on the 1D CNN’s accuracy

**Fig. 7:**
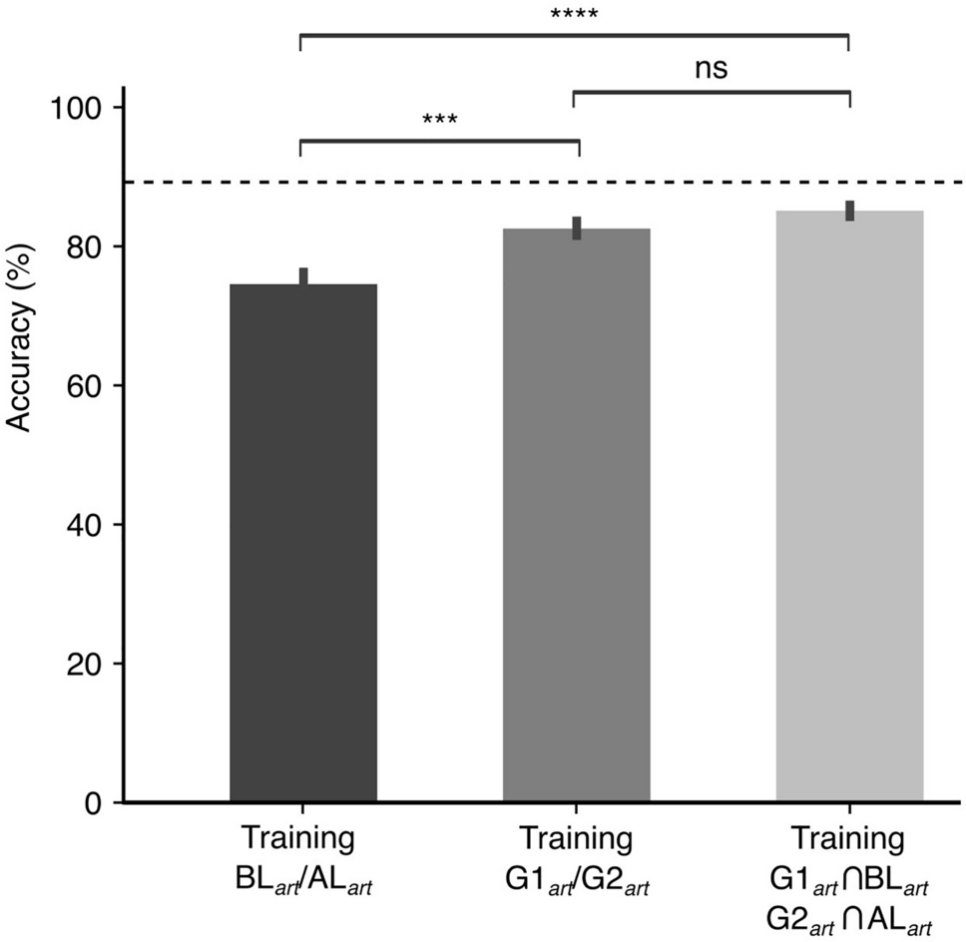
Accuracy comparison for the 1D CNN model using the ${SWR}_{art}$ dataset under three different labeling conditions: training with $\hbox {BL}_{art}$/$\hbox {AL}_{art}$ labels which are created by mixing 20% of Class 1 and Class 2, training with labels after SSL re-labeling ($\hbox {G1}_{art}$/$\hbox {G2}_{art}$), and training on the intersection of $\hbox {G1}_{art}$ with $\hbox {BL}_{art}$ and $\hbox {G2}_{art}$ with $\hbox {AL}_{art}$, with all tests performed against $\hbox {BL}_{art}$/$\hbox {AL}_{art}$. The dashed line represents the accuracy of the 1D CNN model in an ideal context with no label noise. Statistical significance, determined via t-tests, is marked directly above the bars: ****$p> 0.0001$, ***$p> 0.001$ and ‘ns’ for non-significance with $p>0.05$. Vertical lines indicate the standard error of the mean (SEM).

We scrutinized the impact of different level of label noise on the classification performance of the 1D CNN model, both before and after applying SSL re-labeling, utilizing the ${\text{SWR}}_{art}$ dataset. The model’s initial training and validation on the ${\text{SWR}}_{art}$ dataset belonging to Class 1 and Class 2 served as a reference level corresponding to “label noise = 0”, resulted in a baseline accuracy of 87.11% (Fig. 7, dashed horizontal line). This value specifies the model’s potential classification capability in an optimal labeling environment.

The introduction of label noise of the level equal to 20% resulted in a diminution of the accuracy to 73.23% (Fig. 7, left bar c.f. with horizontal dashed line). When SSL re-labeling was used to the noise-affected dataset, the accuracy was significantly improved to 82.16% (N = 5, t-test, $p>0.001$), demonstrating the effectiveness of the SSL re-labeling in mitigating the adverse effects of label noise (Fig. 7, middle bar). Consistent with prior analyses, this study also explored the intersections of $\hbox {G1}_{art}$ with $\hbox {BL}_{art}$ instances and $\hbox {G2}_{art}$ with $\hbox {AL}_{art}$ instances. Upon training the 1D CNN model using this intersected dataset, an accuracy of 83.91% was achieved which was also significantly higher than the accuracy obtained with $\hbox {BL}_{art}$/$\hbox {AL}_{art}$ (N = 5, t-test, $p>0.0001$) (Fig. 7, right bar). Furthermore, the training on the intersected groups ($\hbox {G1}_{art} \cap \hbox {BL}_{art}, G2_{art} \cap AL_{art}$) resulted in a similar level of performance as the training on $\hbox {G1}_{art}$ and $\hbox {G2}_{art}$.

### Robustness of SSL re-labeling method against label noise

**Fig. 8:**
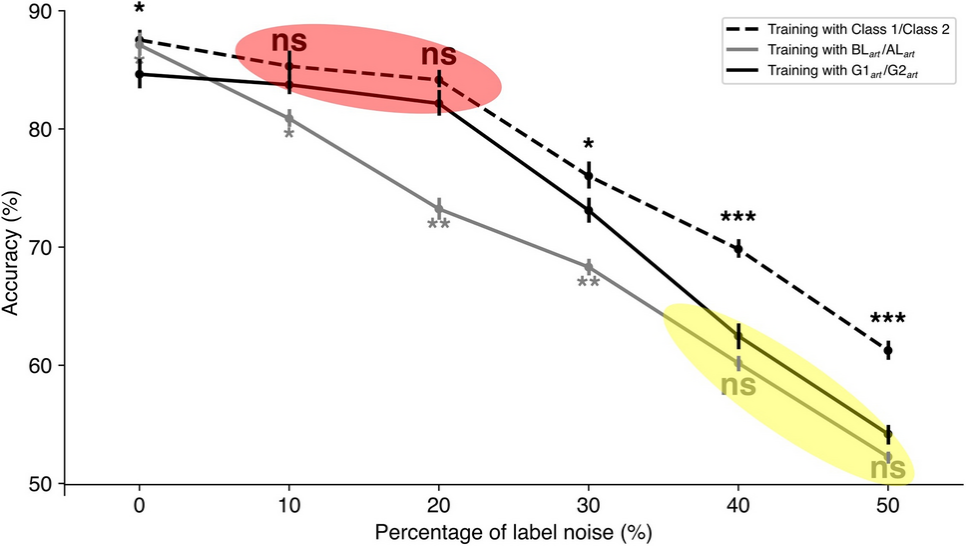
Impact of SSL re-labeling on 1D CNN model training at different levels of label noise. 1D CNN model’s classification accuracy is shown as the percentage of label noise increases (gray solid line), alongside the restorative effects of SSL re-labeling (black solid line) as well as a noise-free dataset (black dashed line) applied during 1D CNN training. SSL re-labeling is most effective at label noise levels between 10% and 20%, where it nearly matches the ideal scenario’s performance (red ellipse). SSL re-labeling is not efficient as label noise approaches 40% and 50% (yellow ellipse). Statistical significance, determined via t-tests, ***$p>0.001,$ **$p>0.01,$ *$p>0.05,$ and ‘ns’ indicates non-significance with $p>0.05$. Vertical lines indicate the standard error of the mean (SEM).

We sought to examine the robustness of our SSL re-labeling method against different degrees of label noise. The experiment’s intent was to observe the impact of progressively introduced label noise on the 1D CNN’s classification accuracy and to test the effectiveness of SSL re-labeling in counteracting this noise. Figure 8 provides a visual representation of this evaluation.

The black dashed trace represents an ideal scenario where the model is trained on a dataset without any label noise ($\hbox {BL}_{art}$ equal to Class 1, $\hbox {AL}_{art}$ equal to Class 2), capturing the upper-bound performance that the model could achieve. The decay of this curve is due to the fact that the model is tested on the $\hbox {BL}_{art}$ and $\hbox {AL}_{art}$ that contain increased level of label noise (0 to 50%). By contrast, the gray solid trace reflects the performance of the 1D CNN model both trained and tested on $\hbox {BL}_{art}$ and $\hbox {AL}_{art}$ containing label noise (0 to 50%). As the noise level increases, the model’s ability to classify correctly diminishes, which is expected since the mislabeled data provides inconsistent signals for model learning and testing.

Interestingly, the black solid trace illustrates the alteration of the accuracy score due to the SSL re-labeling process. At 0% noise, 1D CNN model trained without any label noise performed better than SSL. (Similar results was already shown in Eldele et al. 2021 comparing supervised learning with SSL performance). At noise levels ranging from 10% to 20%, the enhancements of the performance due to the SSL relabeling were significant and nearly approaching the performance of the ideal model (Fig. 8, red ellipse). With the presence of the noise level around 30%, the performance of 1D CNN model with SSL re-labeling was still significantly higher than the performance of 1D CNN model alone but already lower than the upper-bound performance. Finally, at noise levels of 40–50%, the enhancements due to SSL were not significant, indicating the limit of the effectiveness of SSL as the label noise increased (Fig. 8, yellow ellipse).

### Cross-dataset validation

To demonstrate the generalizability of our SSL approach beyond SWR classification, we evaluated its performance on two additional physiological time-series datasets: the MIT-BIH Arrhythmia Dataset and the Epileptic Seizure Recognition Dataset. This validation across different physiological signals provides insights into the broader applicability of our method for handling label noise in clinical data (Table 3).

**Table 3:** Performance comparison across different physiological time-series datasets (mean ± std over 5 runs).

Dataset	Clean Data	With 20% Noise	After SSL
SWR	–	73.28% ± 2.42	84.11% ± 1.20
${\text{SWR}}_{art}$	87.11% ± 0.31	73.23% ± 1.74	82.16% ± 2.25
MIT-BIH	98.70% ± 0.12	77.75% ± 2.31	97.00% ± 0.42
Epileptic	98.59% ± 0.15	76.96% ± 2.84	98.02% ± 0.38

For the MIT-BIH arrhythmia dataset, our baseline model achieved 98.70% ± 0.12% accuracy on clean data, reflecting the typically high performance of deep learning models on ECG classification tasks. After introducing 20% label noise to simulate real-world annotation errors, the performance dropped significantly to 77.75% ± 2.31%, demonstrating the substantial impact of label noise on classification accuracy. Applying our SSL method restored the accuracy to 97.00% ± 0.42%, demonstrating effective noise correction while maintaining balanced class distributions.

Similar results were observed with the epileptic seizure recognition dataset, where the baseline accuracy of 98.59% ± 0.15% on clean data decreased to 76.96% ± 2.84% with 20% injected label noise. Our SSL method successfully restored performance to 98.02% ± 0.38%, approaching the clean-data baseline while preserving class balance.

## Discussion

In this study, we present a novel approach to addressing label noise in time-series classification using self-supervised learning (SSL), with primary focus on Sharp Wave Ripple (SWR) classification. Through comprehensive evaluation, we demonstrate the effectiveness of our method in improving classification accuracy while maintaining robustness to label noise. Our results show significant improvements in SWR classification, which we further validated across other physiological time-series data.

The success of our approach in handling label noise stems from its ability to leverage the inherent temporal structure of the data. Recent work by Zhang et al.^12^ has highlighted how SSL can effectively reduce dependence on labeled data while maintaining high performance. Our results align with these findings, showing particularly strong performance in scenarios with moderate levels of label noise (10–30%). This is especially relevant for physiological signal classification, where obtaining accurate labels often requires significant expertise and is subject to inter-observer variability^2^.

Sharp wave ripples are critically important in understanding memory processes, as these high-frequency oscillatory patterns in the hippocampus are linked with memory consolidation^7^. Accurate classification of SWRs before and after learning tasks is essential for characterizing the distinct neural states associated with learning. In our experiments, we used mice navigating a maze to induce spatial learning, aiming to distinguish the SWR patterns generated before and after the learning phase. This classification task is pivotal for elucidating how SWRs differ in these learning states.

Our initial benchmarking of several deep learning models, including MobileNet, EfficientNetB0, VGG16, and ConvNeXt, alongside traditional 1D models like LSTM and 1D CNN, revealed a consistent performance plateau around 69–73% accuracy, highlighting the impact of label noise within our dataset. To address this limitation, we explored several approaches. Genetic algorithms^29^, implemented by encoding labels as chromosomes with fitness functions designed to maximize classification accuracy, achieved only 70.5% accuracy despite multiple generations of evolution. Similarly, our experiments with autoencoders^30^ showed marginal improvement to 71.2% accuracy.

The breakthrough came with the application of SSL to relabel our dataset. By leveraging the intrinsic structure of the data to generate labels^31^, we achieved a marked improvement in classification accuracy from 73.28% to 84.11%. Validation using our synthetic ${\text{SWR}}_{art}$ dataset confirmed the method’s effectiveness in environments with moderate label noise, showing significant improvement up to 30% noise levels. However, the method’s effectiveness diminished as noise levels approached 40–50%, indicating its operational boundaries.

To demonstrate broader applicability, we validated our approach on other physiological time-series datasets. The MIT-BIH arrhythmia dataset showed remarkable improvement from 77.75% to 97.00% accuracy, approaching its clean-data baseline of 98.70%. Similarly, for epileptic seizure detection, our method restored accuracy from 76.96% to 98.02%, nearly matching the original 98.59% performance. These results on structurally different physiological signals reinforce our approach’s robustness.

The significant improvements achieved through SSL highlight its potential to overcome label noise in electrophysiological data analysis. Unlike traditional approaches that often involve substantial manual intervention and potential data loss^5,11^, our method leverages the inherent structure within the data itself, enabling automated correction crucial for complex datasets like SWRs.

From a biological perspective, improving the classification of sharp wave ripples (SWRs) has significant implications for understanding memory processes. SWRs are critical for memory consolidation, and accurately distinguishing between SWRs generated before and after learning tasks can provide valuable insights into the neural mechanisms underlying memory formation and retrieval^7^.

Enhanced classification accuracy can lead to more precise characterizations of these neural events, facilitating a deeper understanding of how different learning experiences affect hippocampal activity and memory consolidation processes. Better classification of SWRs could also aid in the identification of specific patterns associated with different types of memory tasks or cognitive states, contributing to the development of targeted interventions for memory-related disorders. For example, distinguishing SWR patterns in normal versus pathological conditions, such as Alzheimer’s disease, could inform therapeutic strategies and improve diagnostic accuracy^19^.

While our primary focus was on SWR classification, the method’s success with other physiological signals suggests broader applicability within neurophysiological signal processing. Future research should focus on adapting these techniques for specific clinical applications, particularly where label noise poses significant challenges to accurate diagnosis. The integration of domain-specific physiological knowledge into SSL frameworks could further enhance performance, especially for complex signals like SWRs where subtle temporal patterns carry important biological significance.

This work establishes a foundation for more reliable analysis of physiological time-series data, with immediate applications in neuroscience and potential extensions to clinical diagnostics. Our approach demonstrates that by effectively addressing label noise while preserving critical temporal information, we can significantly improve the accuracy of physiological signal classification, particularly for complex neural signals like SWRs.

## Materials and methods

### SWR

The SWR dataset used to support deep learning applications was recorded from the hippocampal CA1 region of wild-type (WT) mice to study spatial memory formation (see materials and methods in (Jura et al. 2019)). Ethical approval for the study was provided by the University of Bordeaux’s ethical committee (protocols A50120159 and A16323), ensuring all animal procedures adhered to the European Guidelines (Directive 2010/63/EU) for the care and use of laboratory animals. The animals were obtained from the Bordeaux University animal facility and housed one per cage in temperature (22$\pm {1}^{\circ }$C) and humidity-controlled (50 ± 10%) conditions under an automatic 12h light/dark cycle. Mice had ad libitum access to food and water prior to the experimental procedure. This study is reported in accordance with the ARRIVE guidelines. Prior data collection, mice were acclimated to their home cages and then equipped with microelectrodes under surgical conditions that met strict welfare standards. The implanted electrodes, interfacing with a high-resolution (40 kHz) 128-channel Plexon recording system, enabled the capture of local field potentials of hippocampus in behaving animals. After two days of habituation in the 8 arms-radial maze the animals underwent a series of behavioral task in the maze for 6 days. Before and after each trial in the maze, electrophysiological recordings were performed to chart changes correlating with learning behaviors. To prepare the data for analysis, the recorded signals were downsampled and SWRs were extracted. A 4th-order Chebyshev Type II filter, complemented by Hilbert transform techniques, facilitated the identification of SWR events, as established in the protocol by (Jura et al. 2019). Finally, 256-ms windows, each containing an individual SWR in the center, were chosen to form non-overlapping RCIs, guaranteeing the independence of samples for subsequent computational evaluation (see methods of (Hsu et al. 2021)). To ensure methodological rigor and prevent potential biases in our deep learning analyses, we carefully constructed a balanced dataset by selecting an equal number of samples from both before learning (BL) and after learning (AL) classes.

### ${\text{SWR}}_{art}$

The ${\text{SWR}}_{art}$ dataset is a synthetic dataset that simulates sharp wave-ripples (SWRs) using customizable sine waves within the 120–200 Hz frequency range^8^ to match real SWR variability. These waves are shaped by Gaussian envelopes and infused with Gaussian noise and random signals from the delta (1–3 Hz) and low gamma (20–40 Hz) bands for realism^8^. Two sets of 20,000 ${\text{SWR}}_{art}$, representing ‘Before Learning’ and ‘After Learning’ stages, exhibit distinct variability in amplitude and frequency, as well as differing noise levels, to reflect changes in neural activity due to learning, providing a controlled test bed for machine learning models’ performance evaluation.

### MIT-BIH arrhythmia dataset

The MIT-BIH arrhythmia dataset is a widely used benchmark for evaluating arrhythmia detection algorithms. This database contains 48 half-hour excerpts of two-channel ambulatory ECG recordings, digitized at 360 samples per second with 11-bit resolution over a 10 mV range. The recordings were collected from 47 subjects studied by the Beth Israel Hospital Arrhythmia Laboratory between 1975 and 1979, comprising both inpatients (60%) and outpatients (40%). The database includes a diverse set of waveforms and artifacts that reflect real-world clinical conditions^32^.

### Epileptic seizure recognition dataset

The epileptic seizure recognition dataset consists of EEG recordings from multiple subjects, preprocessed and structured for seizure detection. The original recordings were sampled at 173.61 Hz for 23.6 s, resulting in 4,097 data points per subject. For our analysis, each recording was segmented into 1-s intervals (178 data points each), creating a balanced dataset of normal and seizure activity. This dataset is particularly valuable for evaluating our method’s performance on high-frequency neurological signals with well-defined event boundaries^33^.

### SSL model and its training

Our study used self-supervised learning techniques inspired by the TS-TCC (Time-Series Representation Learning via Temporal and Contextual Contrasting) framework^26^ to augment the performance of SWR classification models. These techniques are particularly well-suited to the sequential nature of the time-series SWRs obtained from neural recordings. SWRs are subjected to two augmentation techniques: a strong augmentation using permutation-and-jitter, which splits the signal into random segments that are shuffled and jittered, and a weak augmentation applying jitter-and-scale, which adds random variations and scales signal magnitude. These augmentations result in two views of SWRs, which are then utilized in the temporal contrasting module. The temporal contrasting module employs an autoregressive model to condense the past features of one augmented view to predict future timesteps of another augmented view, meaning it forecasts the subsequent points in the sequence based on the observed data in cross-view prediction mode.

Such cross-view prediction mode aims to maximize agreement of the predicted future timesteps against perturbations introduced by strong and weak augmentations, which is key to the model’s ability to understand the temporal relationships within the data. Complementing the temporal contrasting module, the contextual contrasting module seeks to maximize the similarity within contexts of the same sample and minimize similarity among different samples, enhancing the model’s capacity to learn contextual features through a non-linear projection head.

We trained the SSL model for 500 epochs utilizing our dataset, which comprised unlabeled SWR recordings from both before learning and after learning SWRs. The primary objective during training was to minimize the temporal and contextual contrastive loss, which measures how well the model understands temporal relationships within SWRs and then uses them to distinguish between similar and dissimilar SWR contextual patterns. By minimizing this loss, the model becomes more proficient in differentiating between diverse SWR patterns. By the end of the training process, the refined model was employed to generate new labels for our dataset.

### Label generation

Following the SSL model training, we implemented a systematic approach to generate new labels for our SWR dataset. The trained SSL model learns embeddings in a high-dimensional feature space that captures the intrinsic temporal and contextual patterns within the SWRs. These learned representations naturally position similar SWRs closer together in the feature space, while dissimilar SWRs are placed further apart. To convert these learned representations into discrete labels, we applied k-means clustering with k = 2 to the embeddings, corresponding to our binary classification objective. The clustering process resulted in two groups (G1 and G2) based on the natural distribution of features in the embedding space.

### 1D CNN model and its training

Our study employed a custom-designed 1D CNN^25^ to classify RCIs into two categories: those occurring before and those occurring after a learning event. The architecture of this model is structured to sequentially process and extract meaningful features from the input data. The initial stage of the model consists of several convolutional layers. Each layer applies filters, combined with rectified linear unit (ReLU) activation functions and batch normalization, that capture the intricate feature maps hidden in the RCIs. Subsequent to the convolutional stages, MaxPooling layers are included to reduce the dimensionality of the feature maps. This reduction focuses the model on the most salient features, which are crucial for accurate classification. To mitigate the risk of overfitting-where the model performs well on training data but poorly on unseen data-a Dropout layer is incorporated. This layer randomly omits a subset of features during training, which encourages the model to be robust and generalize better to new data. After processing through these layers, the model’s architecture concludes with a Flatten operation that converts the multi-dimensional feature maps into a one-dimensional feature vector. This vector is then fed into a Dense layer, which acts as the classifier. The Dense layer utilizes the rich, extracted features to determine the final classification of each RCI. At the output, a softmax activation function translates the Dense layer’s activations into a probability distribution over the categories, indicating the likelihood of each RCI belonging to the before learning or after learning category.

A 5-fold cross-validation strategy was utilized to train and validate our 1D CNN model. The dataset was divided into five equal parts, with each part serving as the test set in one of five iterations and the remaining data forming the training set. In each iteration, the model was trained for 200 epochs, allowing it to learn from the entire training set multiple times and minimize the impact of data variability. After training, the model was evaluated using the test set, and performance metrics were calculated. These metrics were then aggregated across all folds to assess the model’s overall performance and stability.

### Statistical analysis

To statistically validate the differences in performance between models, we employed the t-test. The p-value less than 0.05 was considered as statistically significant.

### Code

All basic and advanced algorithms used in this work were developed and implemented with custom-written Python scripts (version 3.10), incorporating PyTorch (version 2.1.1) and TensorFlow (version 2.13) for deep learning model development, SciPy (version 1.11.4) for statistical analysis, and Matplotlib (version 3.8.2) for data visualization. All algorithms used in this work were developed and implemented using Python (version 3.10), incorporating PyTorch (version 2.1.1) for deep learning model development, SciPy (version 1.11.4) for statistical analysis, and Matplotlib (version 3.8.2) for data visualization.

## Data Availability

We have made our complete implementation, including data preprocessing scripts, model architectures, publicly available through our GitHub repository https://github.com/Saber-GRAF/ts-ssl-label-noise.
